# Safety and efficacy of danazol in immune thrombocytopenia: a systematic review

**DOI:** 10.1016/j.rpth.2024.102444

**Published:** 2024-05-17

**Authors:** Sangam Shah, Rukesh Yadav, Abhinav Bhattarai, Krishna Dahal, Sunraj Tharu, Sandesh Gautam, Laba Rawal, Sangharsha Thapa, Sanjit Kumar Sah, Sumit Sharma, Krishna Gundabolu

**Affiliations:** 1 Institute of Medicine, Tribhuvan University, Maharajgunj, Nepal; 2 Kathmandu University School of Medical Sciences, Dhulikhel, Nepal; 3 B. P. Koirala Institute of Health Sciences, Dharan, Nepal; 4 Fred and Pamela Buffett Cancer Center, Department of Internal Medicine, Division of Hematology-Oncology, University of Nebraska Medical Center, Omaha, Nebraska, USA

**Keywords:** danazol, Danocrine, hematology, idiopathic thrombocytopenic purpura, immune thrombocytopenia, immune thrombocytopenic purpura, ITP, review

## Abstract

**Background:**

The treatment landscape for relapsed or refractory immune thrombocytopenia (ITP) after corticosteroids is complex.

**Objectives:**

We aimed to assess the efficacy of danazol in treating ITP and evaluate the safety and adverse events following its administration.

**Methods:**

We searched the databases PubMed, EMBASE, and ClinicalTrials.gov for all published studies assessing danazol’s efficacy and safety in treating ITP. The retrieved studies were screened by title and abstract, followed by full-text screening based on the eligibility requirements. The quality assessment was performed using a set of questionnaires. The data were extracted on the descriptive characteristics of the studies and participants, drug dosage, efficacy measures, and adverse effects, and the data were synthesized.

**Results:**

A total of 17 studies consisting of 901 participants were included. The overall response rate is around 61% in this analysis. Among the participants, 315 (34.9%) were men. The age of participants ranged from 16 to 86 years. Danazol combined with other pharmacologic interventions, including all–trans-retinoic acid or glucocorticoids, generated better results. The most common side effects appear to be liver injury and elevation of liver enzymes, weight gain, oligomenorrhea, amenorrhea, and myalgia.

**Conclusion:**

Danazol at low-to-medium doses was well tolerated and succeeded in improving ITP. Danazol therapy may be helpful in the treatment of chronic ITP that is corticosteroid refractory and when corticosteroids or splenectomy (or both) is contraindicated. Danazol can be considered for further research and development in treating primary immune thrombocytopenia.

## Essentials

• Is danazol effective and safe to use in immune thrombocytopenia?
• In this systematic review comprising 901 participants, a substantial fraction (60.6%) responded to danazol.
• Hepatotoxicity with elevated transaminases was the common side effect.
• Danazol is effective in treating immune thrombocytopenia and has fewer adverse effects.

## 1 Introduction

Immune thrombocytopenia (ITP), also known as idiopathic thrombocytopenic purpura, is one of the common causes of thrombocytopenia in adults and is caused by autoantibodies against platelet antigens. Though many patients with chronic ITP are asymptomatic, clinical manifestations in those having symptoms are mucocutaneous bleeding, such as petechiae, purpura, oropharyngeal bleeding, and epistaxis. Less commonly, patients may present with life-threatening hemorrhages like intracranial hemorrhage or gastrointestinal bleeding. The pathogenesis of primary ITP is yet to be understood entirely. Antibody-mediated destruction of platelets leading to their reduced lifespan is still the dominant hypothesis. Other important mechanisms are autoreactive cytotoxic T cells and humoral and cellular autoimmunity directed at megakaryocytes, causing impaired platelet production [1].

Patients with refractory ITP are those whose platelet counts are extremely low, accompanied by bleeding, and who have not responded to at least 2 therapies [2]. These patients do not always have a splenectomy history. Generally, patients with a platelet count of below 30 × 10^9^/L or at risk of hemorrhage get treatment. The initial first line of therapy is glucocorticoids. Intravenous immunoglobulin and platelet transfusion are used in situations of critical bleeding. First-line treatment aims to rapidly increase platelet counts with a higher initial response rate. Relapses are common when corticosteroids are reduced or discontinued; only 10% to 20% of patients can experience long-term remissions. The initial response rate is over 80%, but less than 50% of patients continue to have normal platelet counts after stopping the corticosteroids [3,4].

Splenectomy has the best evidence for changing the course of the disease and resulting in long-term remission of ITP [5,6]. However, splenectomy has unique risks, including surgical complications, encapsulated bacterial infections, and a higher incidence of thrombotic events [5,7]. Newer treatments, including rituximab, thrombopoietin receptor agonists, and fostamatinib, have been employed in the second line, and further, they are expensive and have unpredictable long-term remission rates. Elevated risks of infection or treatment-related malignancies are seen with these drugs [[8], [9], [10], [11], [12]]. An ideal second-line drug for relapsed or chronic ITP would be something that is corticosteroid-sparing with a lower risk of infections, less adverse events, sustained long-term response, and lower cost. A potential candidate is danazol, which is an attenuated androgen.

Danazol is a synthetic steroid approved by the United States Food and Drug Administration for endometriosis, fibrocystic breast disease, and hereditary angioedema. It acts by suppressing the pituitary–ovarian axis by inhibiting pituitary gonadotropin output. Danazol exerts its effects through a variety of mechanisms. Danazol is also well known for its propensity to bind with corticosteroid and sex hormone–binding globulin, increasing their concentration and efficacy. Danazol can also limit IgG-coated platelet clearance by reducing the amount of Fc receptors on the phagocytic cells [13]. Further the different dosage of Danazol have been studied. Therefore, the main objective of our study is to find out the safety and efficacy of danazol monotherapy in refractory chronic or corticosteroid refractory and splenectomy contradicted-ITP.

## 2 Methodology

### 2.1 Search strategy

Using the keywords “immune thrombocytopenic purpura,” “ITP,” or “idiopathic thrombocytopenic purpura” and “danazol,” a systematic search was performed in the online databases PubMed, ClinicalTrials.gov, and EMBASE while the article search was carried out for all articles till date. In addition, the references list of the trials and articles included were also searched to find further reports. The literature search was conducted from June 15, 2022, to June 25, 2022.

### 2.2 Study selection

The full text was screened after the abstract using Microsoft Excel 2013 (Windows version). The concerned authors were contacted via email for those articles with insufficient information. We retrieved all the references from every study for future analysis. The Preferred Reporting Items for Systematic Reviews and Meta-Analysis (PRISMA) were used to report the article.

### 2.3 Inclusion criteria

Studies fulfilling the following criteria were selected:

i) Patient with primary ITP, including newly diagnosed, persistent, chronic, or refractory, with or without splenectomy.
ii) Any study design (randomized controlled trials with double-blinded design/observational studies/case-control studies) on humans reporting clinical outcomes on danazol.
iii) ITP patients who received danazol.

### 2.4 Exclusion criteria

These criteria were used to eliminate studies from consideration.

(i) Study on other experimental animals.
(ii) Studies that did not employ danazol.
(iii) Articles those were not available in English.
(iv) All other forms of articles, such as case reports and conference abstracts.
(v) Articles whose full texts were not available.
(vi) Study involving children.

The other causes of thrombocytopenia (solid tumors, lymphoma, drug-induced ITP, viral infections, systemic disorders like systemic lupus erythematosus, and primary immunodeficiency) were excluded to include studies pertaining only to primary ITP. According to the International Working Group definition, ITP is categorized as newly diagnosed (up to 3 months since diagnosis), chronic (lasting more than 1 year), or persistent (3 to 12 months since diagnosis) [2].

### 2.5 Data extraction

The authors extracted data on a prespecified data extraction sheet in Microsoft Excel consisting of variables: author and year of study, study duration, study phase, sample size, study design, study country, mean age, gender, danazol dosage, adverse reactions, and efficacy measurement.

### 2.6 Quality assessment

Authors A.B. and S.S. performed the quality assessment of the included studies based on the clarity of the study objectives, the study period stated clearly, and the criteria for patient selection, and the study was conducted in multiple centers. Danazol treatment method and dosage were mentioned; baseline equivalence groups were clearly considered; the definition of the primary outcome complete response rate (CRR) or overall response rate (ORR) was defined before the study; there was an adequate follow-up period; adverse reactions were stated, and the limitations of each study were considered. We did not use quality assessment as an exclusion criterion. Individual study questions were responded with “yes” or “no,” and 1 point was awarded for “yes” and 0 points for “no.” A total score was calculated for each study. The quality of the included studies was judged to be fair for a total score of 8 to 10, average for a total score of 5 to 7, and low for a total score of 0 to 4.

### 2.7 Outcome of interest

#### 2.7.1 Efficacy measurement

According to the international consensus, platelet count (PC) responses were examined as follows:

1. CRR: CRR was defined by the attainment of a PC of >100 × 10^9^/L. Three studies defined it as the attainment of PC of >150 × 10^9^/L [[14], [15], [16]]. We harmonized the PC count value of >100 × 10^9^/L for statistical purposes.
2. ORR: ORR was defined as the attainment of PC of >30 × 10^9^/L for the overall response rate.
3. No response (NR): NR was defined as when the PC attained was <30 × 10^9^/L.

### 2.8 Data synthesis

The data on the efficacy measures from the included studies were taken and included in the descriptive summary. The data were summarized using descriptive statistics, including numbers and percentages. The median and IQRs were used to express continuous variables.

## 3 Results

### 3.1 Search results and study selection

PRISMA checklist was closely followed in this systematic review (Supplementary Table S1). The initial literature search retrieved a total of 822 articles. After screening by title and abstract, 228 studies were subjected to full-text screening. Finally, 17 studies that met the eligibility requirements of our study were included in this review. The entire study selection process is displayed in the PRISMA flowchart in the Figure.

**Figure:**
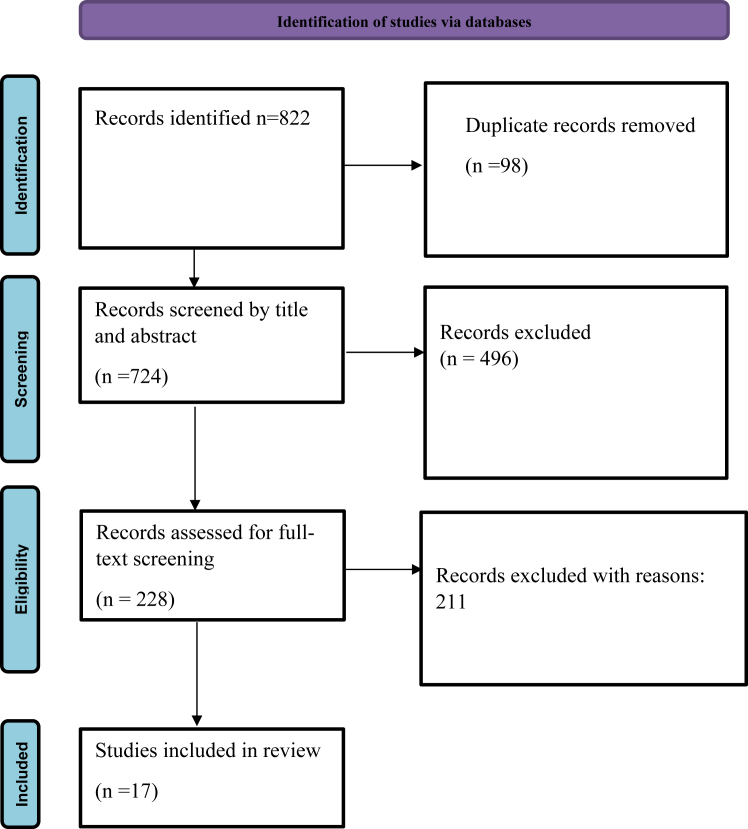
PRISMA flowchart of the included studies. PRISMA, Preferred Reporting Items for Systematic Reviews and Meta-Analysis.

### 3.2 Quality assessment result

In brief, 16 studies were of fair quality, while 1 study was found to possess an average quality. Nine studies scored 10, 5 scored 9, 2 scored 8, and 1 scored 6. The overall score was 8.35, making the overall quality of the included studies fair. The result of the quality assessment of the included studies can be seen in Supplementary Table S2.

### 3.3 Patient characteristics

A total of 17 studies consisting of 901 participants (age, 16-89 years; 315 men) were included. There were 7 studies from Europe, 4 from Asia, and 3 from North America. The number of patients taking danazol was 709, and those taking danazol along with corticosteroids were 204. In addition, 45 of the total participants took danazol combined with all–trans-retinoic acid (ATRA). Based on available data, among 211 patients who had prior splenectomy, 148 received danazol. The descriptive characteristics of the included studies are shown in Table 1 [[14], [15], [16], [17], [18], [19], [20], [21], [22], [23], [24], [25], [26], [27], [28], [29]].

**Table 1:** Descriptive summary and patient characteristics of the included studies.

Author and year of study	Study duration	Stage of trial	Sample size	Study design	Study country	Age (y)	Gender (male:female)	Danazol dosage	Efficacy measures	Adverse reactions in danazol-treated patients
Feng et al., 2017 [18]	12 mo	II	93	Randomized, open-label	China	33.5 (mean)	36:57 population	200 mg	Sustained response, defined as the attainment of a platelet count of ≥30 × 10^9^/L and at least a doubling of baseline platelet count (partial response), or a platelet count of ≥100 × 10^9^/L (complete response), and the absence of bleeding Time to response, duration of response (time to relapse) Peak platelet count	Dry skin, liver injury, headache, dizziness, amenorrhea, hypertension
Liu et al., 2015 [19]	48 mo	NR	319	Retrospective cohort	China	16-86 (range)	96:223 population	200 or 300 mg daily for at least 3 mo	Complete response was defined as an increase in platelet counts to 100 × 10^9^/L and an absence of bleeding. Response was defined as platelet counts 30 × 10^9^/L and ≥2-fold increase in platelet count from baseline, with an absence of bleeding	Liver damage, weight gain, hair loss, acne, hypomenorrhea or amenorrhea, myalgia, hypertension, hyperglycemia, bleeding during sex
Maloisel et al., 2004 [14]	84 mo	NR	57	Prospective	France	53.9 ± 17 (mean ± SD)	23:34	600 mg to 400 mg to 200 mg	Complete response was defined as a platelet count increased to ≥150 × 10^9^/L that was sustained for ≥2 mo; and a partial response was defined as a platelet count increased above 50 × 10^9^/L that was sustained for≥ 2 mo	Increased levels of aspartate or alanine aminotransferase, intracranial hypertension, generalized skin rash
Andres et al., 2003 [16]	108 mo	NR	139	Retrospective analysis	France	43 ± 16 (mean ± SD)	56:83 ratio	mean dosage of 600 mg	Complete response was defined as a platelet count that rose to a normal level (platelet count >150 × 10^9^/L) after treatment for at least 30 d. Partial response was defined as a platelet count of 50-150 × 10^9^/L after treatment for at least 30 d.	Elevation of serum aspartate aminotransferase and alanine aminotransferase level
Edelmann et al., 2015 [20]	31 mo	NR	7	Cross-sectional	Israel	64 (mean)	3:4 population	800 mg/d	Evaluation of response was scaled as follows: 'excellent'—when platelet count rose to ≥100 × 10^9^/L and remained there for at least 2 mo with continued therapy; 'good' - a sustained increase to 50-100 × 10^9^/L; “transient”—when the rise was to more than 50 × 10^9^/L and lasted less than 2 mo; “poor”—when platelet counts did not rise over 20 × 10^9^/L	Headache and nausea
Mylvaganam et al., 1989 [21]	longer than 6 mo duration	NR	15	Cross-sectional	USA	NR	4:11	50 mg/d	The response was defined after 2 mo and classified as follows: excellent when the number of platelets rose to ≥100 × 10^9^/L and remained there for at least 2 mo after discontinuation of therapy; good when platelet count was >50 × 10^9^/L but <100 × 10^9^/L ^3^for ≥ 2 mo; poor when no increase of platelets was achieved.	NR
Buelli et al., 1985 [22]	2 mo	NR	14	Cross-sectional	Italy	51 (Mean)	5:9	200 mg	The response was defined after 2 mo and classified as follows: excellent when the number of platelets rose ≥100 × 10^9^/L and remained there for at least 2 mo after discontinuation of therapy; good when platelet count was> 50 × 10^9^/L but < 100 × 10^9^/L for ≥2 mo; poor when no increase of platelets was achieved.	Weight gain
Ahn et al., 1983 [17]	2 to 13 mo	NR	22	Cross-sectional	USA	51 (Mean)	10:12	200 mg	The response was classified as: Excellent, when the platelet count rose ≥100 × 10^9^/L and remained there for 2 mo or more; good, when the platelet count rose > 50 × 10^9^/L but < 100 × 10^9^/L for ≥2 mo; fair, when platelet count achieved was 20 × 10^9^/L but < 50 × 10^9^/L for ≥2 mo; transient, when platelet count achieved was >n 50 × 10^9^/L but not sustained; and poor, when none of the criteria were met.	Weight gain, weakness, myalgia, headaches, change in menstruation and had spotting, oiliness of the skin, generalized skin eruption
S kotlarek-haus 1987 [23]	9 mo	NR	16	Cross-sectional		34 (mean)	11:5	300 mg	The response was classified as: Excellent; defined as platelet count> 100 × 10^9^/L Good; defined as platelet count (50-100) × 10^9^/L Fair; defined as platelet count >(20-50) × 10^9^/L Insufficient; defined as platelet count <20 × 10^9^/L	Menstrual irregularities, amenorrhea, mild weight gain, hair loss, Elevated liver enzymes
Ambríz, R et al., 1986 [24]	-	NR	25	Cross-sectional		16-41 (range)	0:25	600 mg	Response was Classified as excellent, in which there is a platelet count > 100 × 10^9^/L; (b) good. in which there is symptomatic improvement and the number of platelets rose to >50 × 10^9^/L, but < 100 × 10^9^/L; (c) fair. which made it possible to withdraw prednisone because hemorrhagic manifestations disappeared, even though only a slight increase in platelet level (d) no response. where there was no clinical or laboratory response.	Body weight or the development of acne, asthenia, Myalgias, partial hair loss, and headaches.
Kondo, H et al., 1992 [25]	-	NR	14	Clinical trial	Japan	54 (mean)	11:3	100-400 mg	Excellent response defined by platelets count > 100 × 10^9^/L Good defined by platelets count 50 × 10^9^/L but <100 × 10^9^/L Poor when no increase in platelet was achieved.	NR
Fenaux, P et al., 1990 [15]	48 mo	NR	22	Cross-sectional	France	57 (mean)	12:10	600 mg, 400 mg to child	complete response, normal platelet counts (> 150 × 10^9^/L); partial response: platelet counts between 100 and (50 × 10^9^/L); minor response: platelet counts at least doubled from initial levels, and >40 × 10^9^/L; NR: none of the above.	Weight gain, moderate cholestasis Mild liver function abnormalities
Almagro et al., 1985 [26]		NR	9	NR	Cuba		1:8	400-800 mg	NA	Cephalalgia, overweight, arthralgias, weakness, rash, amenorrhea and breast discomfort, Intracranial bleed
Mazzucconi 1987 [27]		NR	10	NR	Italy	58 (mean)	4:6	600 mg	NA	
Ahn, Y S et al., 1989 [28]	72 mo	NR	96	NR	USA	52 (mean)	36:60	400-800 or 50 mg	Response was classified as: excellent, when the platelet count rose to ≥100 × 10^9^/L and remain ≥2 mo; good, when the platelet count rose to 50 × 10^9^/L but < 100 × 10^9^/L for ≥ 2 mo; fair, when platelet count achieved was 20 × 10^9^/L but <50 × 10^9^/L for ≥2 mo; transient, when platelet count achieved was > 50 × 10^9^/L but not sustained; and poor, when none of the criteria were met.	Weight gain myalgia lethargy skin rash itching hair loss mild virilization liver enzymes elevation
Mcverry, B A et al., 1985 [29]	3 mo	NR	10	NR	UK	60.8 (27-86 [median, (IQR)])	1:9	400-600 mg	Response was classified as: Excellent, when the platelet count rose ≥100 × 10^9^/L and remain there for ≥2 mo; good, when platelet count rose to 50 × 10^9^/L but < 100 × 10^9^/L for ≥2 mo; fair, when platelet count achieved was 20 × 10^9^/L but < 50 × 10^9^/L for ≥2 mo; transient, when platelet count achieved was ≥50 × 10^9^/L but not sustained; and poor, when none of the criteria were met.	Headache nausea
Schiavotto, C et al., 1993 [30]	60 mo	NR	33	Prospective study	Italy	66 (mean)	6:27	400-800 mg	Response was classified as: Excellent, when the platelet count rose to ≥100 × 10^9^/L and remain there for ≥2 mo; good, when platelet count rose to 50 × 10^9^/L but < 100 × 10^9^/L for ≥ 2 mo; fair, when platelet count achieved was 20 × 10^9^/L but <50 × 10^9^/L for ≥2 mo; transient, when platelet count achieved was ≥50 × 10^9^/L but not sustained; and poor, when none of the criteria were met.	Dyspepsia, stroke, weight gain, seborrhea

Age is expressed in mean, mean ± SD, median (IQR), or range.

Gender is expressed in male:female.

NR, not reported.

**Table 2:** Efficacy measurement among the included studies.

Author and year of study	Efficacy measures	Values
Feng et al., 2017 [18]	1. Complete response	ATRA plus danazol: 17/45 (38%) Danazol: 4/48 (8%) or for ATRA plus danazol vs danazol (95% CI): 6.68 (2.04-21.90)
	2. Partial response	ATRA plus danazol: 20/45 (44%) Danazol: 17/48 (35%) or for ATRA plus danazol vs danazol (95% CI): 1.46 (0.63-3.36)
	3. No response	ATRA plus danazol: 8/45 (18%) Danazol: 27/48 (56%) or for ATRA plus danazol vs danazol (95% CI): 0.17 (0.065-0.44)
	4. Peak platelet count	ATRA plus danazol: 178 × 10^9^/L (62-192) Danazol: 69 × 10^9^/L (47-94) or for ATRA plus danazol vs danazol (95% CI): 5.58 (1.73-19.21)
	5. Duration of response (d)	ATRA plus danazol: 18.2 (9-20) danazol: 10.5 (6-12) or for ATRA plus danazol vs danazol (95% CI): 3.10 (2.23-10.75)
Liu et al., 2015 [19]	1. Complete response	Patients received danazol alone: 27/103 (26.2%) Patients received danazol and GCs: 50/191 (26.2%)
	2. Overall response	Patients received danazol alone: 65/103 (63.1%) Patients received danazol and GCs: 126/191 (66.0%)
	3. Platelet count	Patients received danazol alone: 1. Before danazol: 22 × 10^9^/L 2. After danazol: 50 × 10^9^/L Patients received danazol and GCs: 1. Before regimen: 23 × 10^9^/L 2. After regimen: 53 × 10^9^/L
	4. Duration of response (mo)	Patients received danazol alone: 1.5 (0.5-29.0) Patients received danazol and GCs: 1.0 (0-15.0)
Maloisel et al., 2004 [14]	1. Complete response	9/57 (16%)
	2. Partial response	NR
	3. Platelet count	Before danazol:13 ± 13 × 10^9^/L After danazol: 142 ± 100 × 10^9^/L 10 /L
	4. Overall response rate	38/57 (67%)
	5. Median duration of remission	119 ± 45 mo (range, 3 to 182 mo)
Andres et al., 2003 [16]	1. Complete response	1. Corticosteroid:61/118 (52%) 2. Splenectomy:48/55 (87%) 3. Danazol:10/33 (30%)
	2. Partial response	1. Corticosteroid:36/118 (31%) 2. Splenectomy:3/55 (5%) 3. Danazol:14/33 (42%)
	3. Platelet count	Before danazol: 20 ± 13 × 10^9^/L Range (0-147) × 10^9^/L After danazol: NA
	4. Mean duration of response	1. Corticosteroid: 5.2 ± 1.3 mo (range, 4 d to 7 y) 2. Splenectomy: 13.5 ± 11 mo (range, 2-56) 3. Danazol: 84 mo
Edelmann et al., 2015 [20]	1. Excellent result	3/7 (42%)
	2. Good result	1/7 (14%)
	3. Transient result	1/7 (14%)
	4. Platelet count	Before danazol: 20 × 10^9^/L (9-50) After danazol: 87.3 × 10^9^/L (7-209)
Mylvaganam et al., 1989 [21]	1. Excellent	3/15 (20%)
	2. Good	4/15 (26.7%)
	3. Fair	3/15 (20%)
	4. Poor	5/15 (33.33%)
	Platelet count	Before danazol: 32.2 ± 19.6 × 10^9^/L After danazol: At 1 mo: 4-130 × 10^9^/L At 3 mo: 5-150 × 10^9^/L
Buelli et al., 1985 [22]	Excellent:	5/14 (35.7%)
	Good:	2/14 (14.2%)
	Poor:	7/14 (50%)
	Platelet count	Before danazol: 43.14 × 10^9^/L (20-65) After danazol: 84.64 × 10^9^/L (11-150)
Ahn et al., 1983 [17]	Excellent	11/22 (50%)
	Good	2/22 (9%)
	Fair	2/22 (9%)
	Transient	3/22 (13.6%)
	Poor	4/22 (18.1%)
	Platelet count	Before danazol:( 7-87)×1 10^9^/L (30.3 × 10^9^/L) After danazol: 10-286 × 10^9^/L (164.6 × 10^9^/L)
S Kotlarek-Haus 1987 [23]	Excellent	3/16 (18.75%)
	Good	2/16 (12.5%)
	Fair	1/16 (6%)
	Insufficient	5/16 (31%)
	Platelet count	Before danazol:21.2 × 10^9^/L (1-60) After danazol: 54.7 × 10^9^/L (4-170)
Ambríz, R et al., 1986 [24]	Excellent	12/25 (48%)
	Good	NA
	Fair	9/25 (36%)
	No	4/25 (16%)
	Platelet count	Before danazol: 60 × 10^9^/L (1-60) After danazol: 102 × 10^9^/L (25-321)
Kondo, H et al., 1992 [25]	Excellent	8/14 (57%)
	Good	3/14 (21%)
	Poor	3/14 (21%)
	Platelet count	Before danazol: NA After danazol: NA
Fenaux, P et al., 1990 [15]	Complete response	2/22 (9%)
	Partial response	1/22 (4%)
	Minor response	4/22 (18%)
	No response	15/22 (68%)
	Platelet count	Before danazol: 23.4 × 10^9^/L (2-50) After danazol: 48.2 × 10^9^/L (3-202)
Almagro1985 [26]	Platelet count	NR
	Good response	1/9 (11.11%)
	Transient response	1/9 (11.11%)
	No response	7/9 (77.77%)
	
Mazzucconi 1987 [27]	Platelet count	Before: 24 × 10^9^/L After:8-69 × 10^9^/L
	Good response	0/10 (0%)
	Transient	1/10 (10%)
	No response	9/10 (90%)
Ahn, Y S et al., [17]	Overall response (excellent and good)	57/96 (61.4%)
	Platelets count	Before 36 ± 24 × 10^9^/L After 145 ± 77 × 10^9^/L
McVerry, B A et al.,1985 [29]	Good response	1/10 (10%)
	Transient response	2/10 (20%)
	No	7/10 (70%)
	Platelets	NA
Schiavotto, C et al., 1993 [30]	Platelet count	Before: 8 (2-61) × 10^9^/L After: 8-69 × 10^9^/L
	Response (not specified)	10/17 (56%) (*P* value not significant)
	Time to response (d)	30 (7-90)

### 3.4 Adverse effects

In the study by Feng et al. [18], only 1/48 (2%) patients on danazol monotherapy developed grade III liver injury. No fatality attributable to therapy or an adverse event of grade 4 or worse occurred [18]. Sixty-eight of 319 (21.1 %) patients overall had mild or moderate side effects (grades I and II), while 4/319 (1.2%) patients stopped taking their medication because of severe adverse effects (grades III and IV), including liver damage (*n* = 1), hyperglycemia (*n* = 1), and amenorrhea (*n* = 2). Liver injury was the most common adverse effect (*n* = 36). The most frequent harm was a mild or moderate increase in aspartate or alanine aminotransferase [19]. Patients who received danazol for longer than 6 months had a higher incidence of liver function abnormalities (*P* = .048). Danazol dosage reduction or a combination of liver protection medications may be used to restore normal liver function.

However, 9/57 patients (16%) in the study by Maloisel et al. [14] discontinued danazol therapy due to significant side effects, including elevated aspartate or alanine aminotransferase levels (*n* = 5), intracranial hypertension (*n* = 2), generalized skin rash (*n* = 1), and rhabdomyolysis (*n* = 1). ATRA was used in combination with danazol in this study. Only mild to moderate adverse effects were observed in 20/57 patients, indicating that most patients had good tolerance to the medication, the most common side effect being weight gain, edema, and liver test abnormalities [14].

Few studies reported no side effects, which might be attributable to a lower dose of danazol therapy [21,25]. In all the remaining studies, documented tolerable side effects were rapidly reversible with the discontinuation of danazol. The most common side effects were liver injury and elevation of liver enzymes, weight gain, oligomenorrhea, amenorrhea, and myalgia. The adverse effects observed in the studies have been listed in Table 1. However, some rare but serious adverse effects were observed, such as intracranial hypertension, intracranial hemorrhage, or rhabdomyolysis [14,18]. The degree and frequency of adverse effects diminished while the medication was prolonged; side effects also resolved when the drug was withheld or its dose was reduced.

### 3.5 Efficacy

Overall, a substantial fraction of participants observed an excellent response to danazol. However, there were many participants with no satisfactory response as well. Studies suggested that danazol combined with other pharmacologic interventions, including ATRA or glucocorticoids, generated better results. Feng et al. [18] showed that administration of danazol along with ATRA produced a complete response in a more significant number of participants (38%) than that of danazol alone (8%, OR [95% CI], 6.68 [2.04-21.90]; *P* = .00098). In the same study, ATRA combined with danazol significantly raised the PC compared with danazol alone (OR [95% CI], 5.58 [1.73-19.21]; *P* = .01). When danazol was combined with glucocorticoids (GCs), there were no significant differences in the response in PC compared with danazol alone. However, the time of achievement of response was comparatively faster with the incorporation of GCs than that with danazol alone.

Overall response: 430/709 (60.6%).

No response: 279/709 (39.4%). The details of the response is shown in Table 2.

## 4 Discussion

For more than 35 years, danazol has been used as a second-line treatment for ITP [17]. Overall, a significant fraction of participants attained an excellent response (platelets count above 100 × 10^9^/L) to danazol. Studies suggested that danazol combined with other pharmacologic interventions, including ATRA or glucocorticoids, generated better results. Even low-dose danazol resulted in good results with fewer side effects. Even after the corticosteroid dose was decreased, a low–medium dose of danazol effectively kept the PC high. Moreover, the patients were able to sustain remission after a long-term danazol therapy.

Among 96 patients, Ahn et al. [17] reported an OR rate of 61.4%. Maloisel et al. [14] conducted a prospective study on 57 patients with chronic ITP, with 67 percent acquiring OR and 16 percent achieving CR. In a multicenter, randomized, open-label, phase 2 study done in China by Feng et al. [18], patients receiving ATRA plus danazol had a higher rate of sustained response (defined as the attainment of a PC of 30 × 10^9^/L or more) than those getting danazol alone: 28 (62%) of 45 vs 12 (25%) of 48 (odds ratio, 4.94; 95% CI, 2.03-12.02; *P* = .00037) at the 12-month follow-up. In the same study, patients receiving danazol monotherapy exhibited a poorer sustained response than expected, presumably due to the disease’s refractoriness and the comparatively modest dose and duration of danazol. Few studies, however, have revealed inconsistent results with low responses [15,26,27]. These disparities could be attributed to variances in sample sizes, research populations, and therapy techniques. Danazol’s response is influenced by gender, age, and spleen status. The disparities in results could be attributed to differences in patient populations or the small number of patients studied. Furthermore, in all 3 reports with no significant efficacy, the duration of therapy was limited to 2 to 3 months.

Danazol is less expensive than rituximab and TPO-receptor agonists.

It has been demonstrated that immunosuppressive medications such as vinca alkaloids, cyclophosphamide, and azathioprine can increase PCs in some people. In the recently published FLIGHT study, which included both primary and secondary ITP patients, the addition of mycophenolate mofetil in first-line treatment to glucocorticoids improved response rates with lower risk of relapse or refractory ITP at the expense of poorer quality of life [31]. The short-lived effects of vinca alkaloids, the carcinogenic risk of cyclophosphamide, and the sterility and bone marrow–suppression side effects are substantial drawbacks. In addition to having operational and postoperative risks, splenectomy is not beneficial for many individuals. The efficacy of high-dose intravenous immunoglobulin IgG is only transient. The options for second-line therapy for individuals with primary immune thrombocytopenia can include splenectomy, rituximab, thrombopoietin receptor agonists, and other drugs like immunosuppressants or immunomodulators. Eltrombopag has a response rate of 59% to 88%, with a median time to response of 1 to 4 weeks, but there is an increased risk of hepatotoxicity. Romiplostim has a similar efficacy of around 71% to 88% [11,[32], [33], [34]]. About 57% to 62.5% of patients respond to rituximab; the average response time is 4 to 8 weeks [10,35,36]. According to our findings, the overall response to danazol monotherapy was 59.17%, comparable to other second-line therapies for ITP. The use of danazol in low-income countries can be pivotal as the cost of danazol is comparatively lower than that of other therapeutic options for ITP, like azathioprine, corticosteroids, rituximab, and splenectomy. Furthermore, danazol’s safety profile and availability are also better than those of the abovementioned treatment.

Danazol, an attenuated androgen, may have an immunomodulatory effect on immune disorders like ITP. Studies have shown that danazol has a corticosteroid-sparing effect such that it reduces the dose and can replace them once remission of ITP is achieved [17,24]. Similarly, when used as a combination therapy, the side effects of steroids can be minimized to a significant extent as danazol allows to reduce the dose of the steroids [25]. According to Barbieri et al. [37], danazol can bind to steroid-binding globulin and improve its accessibility to other tissues by displacing and liberating active hormones. The synergistic activity of glucocorticoid and danazol allowed for glucocorticoid tapering, which can reduce the adverse effects of high-dose glucocorticoid therapy.

This study supported the efficacy of low-dose danazol. Danazol’s recommended daily dose ranges from 400 to 800 mg, with a response time of about 2 to 3 months. Patients receiving danazol monotherapy exhibited a poorer sustained response than expected, presumably due to the disease’s refractoriness and the comparatively modest dose and duration. However, the likelihood of side effects increases with long-term treatment duration and higher doses. Acne, increased facial hair, unbalanced cholesterol metabolism, reduced liver function, and atypical menorrhea are common adverse effects. When given at higher doses (400-800 mg/d) for numerous years, danazol may have been linked to hepatic adenomas and cancer [38,39]. Several studies used a lower danazol dose (50-400 mg/day) to minimize side effects and promote better tolerability among patients. The response rates in most patients were comparable with those reported with the standard dose.

Our study had certain limitations. We could not assess the efficacy or adverse effects differentiated by gender or spleen status as the studies did not have stratified analysis. Yet, the study could provide practical recommendations for clinicians considering the use of danazol in managing ITP patients. Furthermore, due to a lack of studies, we could not explain the role of danazol for ITP patients in low-income countries. Hence, this study also warrants the need for large-scale multicenter randomized control trials with a more extended follow-up period to assess the efficacy and safety of danazol in the treatment of ITP.

## 5 Conclusion

Danazol combined with other pharmacologic interventions, including ATRA or glucocorticoids, has generated better results based on our study. However, it could cause more severe adverse effects as well. Danazol at low-to-medium doses was well tolerated and seemed effective in relapsed or refractory ITP. Danazol therapy may be helpful in the treatment of chronic ITP that is corticosteroid refractory and when corticosteroids or splenectomy (or both) are contraindicated. Further research is warranted to investigate the role of danazol in treating primary immune thrombocytopenia. In addition, different combinations of immune-suppressing agents, including danazol, should be investigated to identify additional approaches to achieve a sustained response in patients with primary immune thrombocytopenia.
